# Test-retest reliability of functional near-infrared spectroscopy during a finger-tapping and postural task in healthy older adults

**DOI:** 10.1117/1.NPh.10.2.025010

**Published:** 2023-05-26

**Authors:** Veerle de Rond, Moran Gilat, Nicholas D’Cruz, Femke Hulzinga, Jean-Jacques Orban de Xivry, Alice Nieuwboer

**Affiliations:** a KU Leuven, Department of Rehabilitation Sciences, Neurorehabilitation Research Group, Leuven, Belgium; b Leuven Brain Institute, Leuven, Belgium; c KU Leuven, Department of Kinesiology, Motor Control and Neuroplasticity Research Group, Leuven, Belgium

**Keywords:** Functional near-infrared spectroscopy, ageing, test-retest, reliability, postural control, finger tapping

## Abstract

**Significance:**

Functional near-infrared spectroscopy (fNIRS) is increasingly employed in studies requiring repeated measurements, yet test-retest reliability is largely unknown.

**Aim:**

To investigate test-retest reliability during a postural and a finger-tapping task with and without cap-removal.

**Approach:**

Twenty healthy older adults performed a postural and a finger-tapping task. The tasks were repeated twice in one session and once the next day. A portable fNIRS system measured cortical hemodynamics (${\mathrm{HbO}}_{2}$) in five regions of interest for the postural task and in the hand motor region for finger-tapping.

**Results:**

Test-retest reliability without cap-removal was excellent for the prefrontal cortex (PFC), the premotor cortex (PMC) and the somatosensory cortex (SSC) (intraclass correlation coefficient (ICC)≥0.78), and fair for the frontal eye fields (FEF) and the supplementary motor area (SMA) (ICC≥0.48). After cap-removal, reliability reduced for PFC and SSC (ICC≥0.50), became poor for SMA (ICC=0.01) and PMC (ICC=0.00) and remained good for FEF (ICC=0.64). Similarly, good reliability (ICC=0.66) was apparent for the hand motor region without cap-removal, which deteriorated after cap-removal (ICC=0.38).

**Conclusions:**

Test-retest reliability of fNIRS measurements during two separate motor tasks in healthy older adults was fair to excellent when the cap remained in place. However, removing the fNIRS cap between measurements compromised reliability.

## 1 Introduction

Functional near-infrared spectroscopy (fNIRS) is a neuroimaging technique that uses the principle of neuro-vascular coupling to estimate the blood oxygen-level dependent (BOLD) response as a surrogate for neural activation and deactivation.^1^ Brain oxygenation is measured through light-emitting and receiving optodes using two different wavelengths within the “optical window” of 700 to 900 nm differentiating between oxygenated (${\mathrm{HbO}}_{2}$) and deoxygenated hemoglobin (HHb) absorption.^2^^,^^3^ fNIRS is a non-invasive and user-friendly technique, which has been validated against functional magnetic resonance imaging.^4^ Among the drawbacks of fNIRS are its limited spatial resolution and cortical penetration.^5^ However, fNIRS systems are mobile and less sensitive to motion artifacts than other mobile neuroimaging systems, such as electroencephalography (EEG),^6^ allowing for measurements during actual whole-body movements in natural environments.^7^

For these reasons, fNIRS is becoming increasingly popular for investigating the cortical activation patterns during postural and gait-related tasks.^5^^,^^8^ In addition, more and more intervention studies are implementing fNIRS as a primary outcome, comparing cortical activation before and after training in repeated measures designs.^9^^–^^13^ For example, studies have used fNIRS to investigate learning-induced neural changes in the prefrontal cortex (PFC) as well as in other motor regions during balance^9^^,^^12^^,^^13^ and manual tasks^10^^,^^11^ in young adults. These studies, however, did not take test-retest reliability into account, crucial for interpreting the changes in the BOLD signal as being meaningful rather than signifying measurement error. There are several potential sources of error, including the limited spatial specificity of fNIRS,^14^ as well as the systemic changes in physiological measures that may arise during movement due to heart rate and blood flow alterations.^15^ Other possible inaccuracies may arise from day-to-day variability in hemodynamic oscillations and whether the fNIRS-cap was repositioned precisely by the operator.^16^

So far, fNIRS test-retest reliability has shown to be fair to excellent during resting-state.^17^^–^^19^ Seven studies investigated fNIRS test-retest reliability of the PFC or the contralateral motor cortex during motor tasks, based on the intraclass correlation coefficient (ICC).^20^^–^^24^ The ICC provides an overall estimate of the correlation and agreement between measurements. It is calculated as the proportion of between-subject variance over the total variance.^25^ Four studies reported good to excellent reliability during manual tasks (ICC≥0.60), of which two studied task-specific motor channels (ICC≥0.62).^20^^,^^22^ One study reported very poor test-retest reliability (ICC=0.002) during passive hand movements imposed by a robot.^21^ Only two studies investigated test-retest reliability during gait-related tasks,^26^^,^^27^ showing fair to good reliability for straight walking (ICC>0.40;^26^ ICC=0.71^27^) and turning (ICC=0.67^27^) in the PFC in young and middle-aged adults. None of these studies were conducted in older adults, who may present lower signal to noise ratios.^28^ Two studies pertained to patients with multiple sclerosis^26^ and traumatic brain injury,^23^ resulting in poor (ICC<0.40) and good (ICC=0.70) reliability in the PFC.

Given the above-described gaps in the literature, we set out to investigate the test-retest reliability of fNIRS in healthy older adults during two different motor tasks, namely a postural weight-shifting and a finger tapping task. First, test-retest reliability of five regions of interest (ROIs) was determined during a weight-shifting task comparing two tests, which were repeated twice on the same day and on two consecutive days following cap removal and repositioning. Next, the task-specificity of test-retest reliability was investigated during a control finger tapping task, focusing on the contralateral finger region of the motor cortex.^29^ Based on earlier work during both gross and fine motor tasks in young adults^26^^,^^27^ and the anticipated lower signal to noise ratios in older people,^28^ we hypothesized that test–retest reliability would be more compromised than that obtained in young adults. Furthermore, we expected lower reliability after cap removal compared to when the cap remained stationary.

## 2 Materials and Methods

### 2.1 Participants

Healthy older adults were recruited via an existing local database, compliant with the general data protection regulation (GDPR), as part of a larger randomized controlled trial (RCT; clinicaltrial.gov ID: NCT04594148). Participants had to be at least 65 years old, right handed (self-reported) and be able to independently stand upright for at least 5 min. Participants were excluded if they had a self-reported history of neurological disorders, balance impairments (i.e., vestibular disorders), uncorrected visual impairment, chronic musculoskeletal problems (e.g., osteoarthritis, osteoporosis), cardiovascular (e.g., uncontrolled hypertension, peripheral vascular disease) or respiratory (e.g., chronic obstructive pulmonary disease) disease, or diabetes related polyneuropathy. Additionally, participants with a cognitive impairment (Montreal cognitive assessment (MoCA)<26) were excluded. Written informed consent was obtained from all participants prior to enrolment. The study was approved by the Ethics Committee Research UZ/KU Leuven (study ID: S62917).

### 2.2 Experimental Procedure and Tasks

#### 2.2.1 Procedure

Only participants who were randomized to the passive control group of the RCT, who received no intervention, were included. Participants were assessed on 2 consecutive days. Motor tests were assessed twice on day 1, before and after a resting period of 30 min while keeping the fNIRS cap and optodes in place. After a complete removal of the fNIRS system, the cap was re-attached the next day and the third test was conducted around the same time of day as the first measurement the day before. Cap position across days was standardized (see Sec. 2.2.4). Motor and fNIRS assessments were conducted using a block design with each block consisting of seven trials of 20 sec of rest followed by 20 sec of movement. Prior pilot testing showed an 80% true positive rate when using this design. The start of each trial was marked in the fNIRS signal with a task-synchronized trigger. The 20-sec resting period, conducted in stance for the postural task and in sitting for the tapping task, served as a baseline for the following movement trial. The postural task blocks were conducted before the tapping task in a fixed order.

#### 2.2.2 Postural task

The postural task consisted of a non-immersive virtual reality weight-shifting task,^30^ as described in detail elsewhere (see Figure S1 in the Supplementary Material).^31^ In short, participants were standing in front of a screen at approximately three meters distance. They were instructed to shift their weight mediolaterally >80% of their a-priori determined individual limits of stability, which activated a virtual water jet. With the water jet, they attempted to hit as many virtual wasps as possible, which appeared on the left and right side of the screen alternately. Participants’ center of mass (CoM) was captured within Nexus software (Vicon, Oxford Metrics, United Kingdom) by recording reflective markers placed bilaterally on the acromia, posterior superior iliac spines, lateral epicondyles, and lateral malleoli. Calculation of the CoM was done online within the D-Flow software (Motek Medical BV, Amsterdam, The Netherlands; version 3.28), based on the formulation by Winter (2009).^32^ During rest, subjects were asked to stand and watch the screen, displaying a video recording of the wasp game.

#### 2.2.3 Finger tapping task

The finger-tapping task, as part of the cloud-UPDRS application (version 1.3.0),^33^ was performed with the right dominant hand on a smartphone. It consisted of two visual targets with a diameter of 0.6 inch, positioned at a 2-inch distance from each other (see Figure S2 in the Supplementary Material). Participants were seated on a chair in front of the smartphone placed on a table. They were instructed to alternately tap the left and right targets with their right index finger while holding the smartphone still with their left hand. To prevent any learning effect, tapping frequency was set at 180 beats per minute imposed by a metronome beat. The researcher verbally indicated the start and termination of the task. Participants were instructed to sit as still as possible and place their right hand flat on the table during the 20-sec resting periods in between tapping trials.

#### 2.2.4 fNIRS assessment

Brain hemodynamics were recorded following recent consensus guidelines.^5^ A continuous wave, single-phase fNIRS system (NIRSport2, NIRx, Berlin, Germany), using light emitting diodes (LEDs) with wavelengths of 760 and 850 nm at a sampling frequency of 7.81 Hz, recorded brain hemodynamics within the Aurora software (version 2020.7). First, participants’ head circumference was measured and matched to the closest corresponding cap size, varying from 54 to 60 cm. The Cz anatomical landmark was then determined using the inion, nasion, and pre-auricular points after which the lightweight cap was carefully placed on the head. To ensure similar cap placement on day two, the Cz, CP2, and FC1 anatomical landmarks were marked. Participants were asked not to wash their hair in between measurement days.

A total of 32 optodes (16 sources, 16 detectors) were used to cover predefined ROIs, including the prefrontal cortex (PFC; Brodmann areas 9, 10, and 46), frontal eye fields (FEF; Brodmann area 8), supplementary motor area (SMA; Brodmann area 6, medial), premotor cortex (PMC; Brodmann area 6, lateral), and somatosensory cortex (SSC; Brodmann areas 1, 3, 5 and 7). The 10-10 layout in the fNIRS Optodes’ Location Decider (fOLD) toolbox^34^ was used to specify the optode locations (see Table S1 in the Supplementary Material) with a source-detector separation of ∼3  cm. Sixteen short-separation channels with a source-detector separation of 8 mm were included, one over each source, to correct for physiological noise in the fNIRS signal.^35^ Furthermore, two accelerometers were placed at the back of the cap to correct for movement artefacts related to head movements. At the onset of testing, signal quality was visually checked and improved by moving the hair aside from underneath the optodes, if needed. An additional opaque cap was then placed over the fNIRS set-up to protect external light from interfering with the hemodynamic measurements. For the tapping task, the motor channel in between optode C3-C1 of the contralateral (left) hemisphere was chosen as the ROI. As the fOLD toolbox does not provide specific information on anatomical brain representations, the C3-C1 hand motor channel was based on the EEG topography^36^ in accordance with previous data on the homunculus hand position.^37^

### 2.3 Data Processing

#### 2.3.1 Behavioral data

Weight-shifting data were exported by D-Flow and analyzed within MATLAB 2018b (MathWorks, Natick, Massachusetts, United States). Similar to our previous analysis,^31^ CoM data were first low-pass filtered with a fourth-order Butterworth filter (cut-off = 6 Hz). Weight-shifting was then determined as the movement from the 80% stability limits threshold on the right to the 80% stability limits threshold on the left and vice versa. Outcome measures included weight-shifting speed and accuracy (CoM error) averaged over the seven trials.

The cloud-UPDRS smartphone data were analyzed with Microsoft Excel (version 2016). Outcome measures included the average number of taps per trial and the accuracy of target taps in pixels (target error) and calculated as:

$$\sqrt{(\text{Xposition}-\text{Xtarget}{)}^{2}+(\text{Yposition}-\text{Ytarget}{)}^{2}},$$ (1)

where Xposition and Yposition refer to the coordinates of the finger on the screen, and Xtarget and Ytarget refer to the coordinates of the targets on the screen.

#### 2.3.2 fNIRS data

Brain hemodynamic data were analyzed with the open access NIRS toolbox (https://github.com/huppertt/nirs-toolbox)^38^ implemented in MATLAB 2018b (MathWorks, Natick, Massachusetts, United States). Raw intensity signals were checked visually, resampled to 5 Hz, and converted into optical density using the Beer-Lambert law and a partial path length correction factor of 0.1, thereby correcting for light scatter caused by the brain tissue that the near-infrared light was travelling though, as stated in previous research.^39^^,^^40^ A general linear model, including short separation channels and accelerometers, was used to estimate the task hemodynamic response, thereby correcting for signal variations due to physiological noise and movement artefacts.^20^^,^^35^ The autoregressive iteratively reweighted least squares method was implemented to correct for motion and auto correlated noise.^41^ This method, including short separation channel regression, outperformed other filtering methods^35^ and was shown to improve data reproducibility.^35^ Accelerometers identified and corrected for changes in the fNIRS signal that corresponded with the accelerometer signal over a time-window of 15 s.^42^

**Fig. 1:**
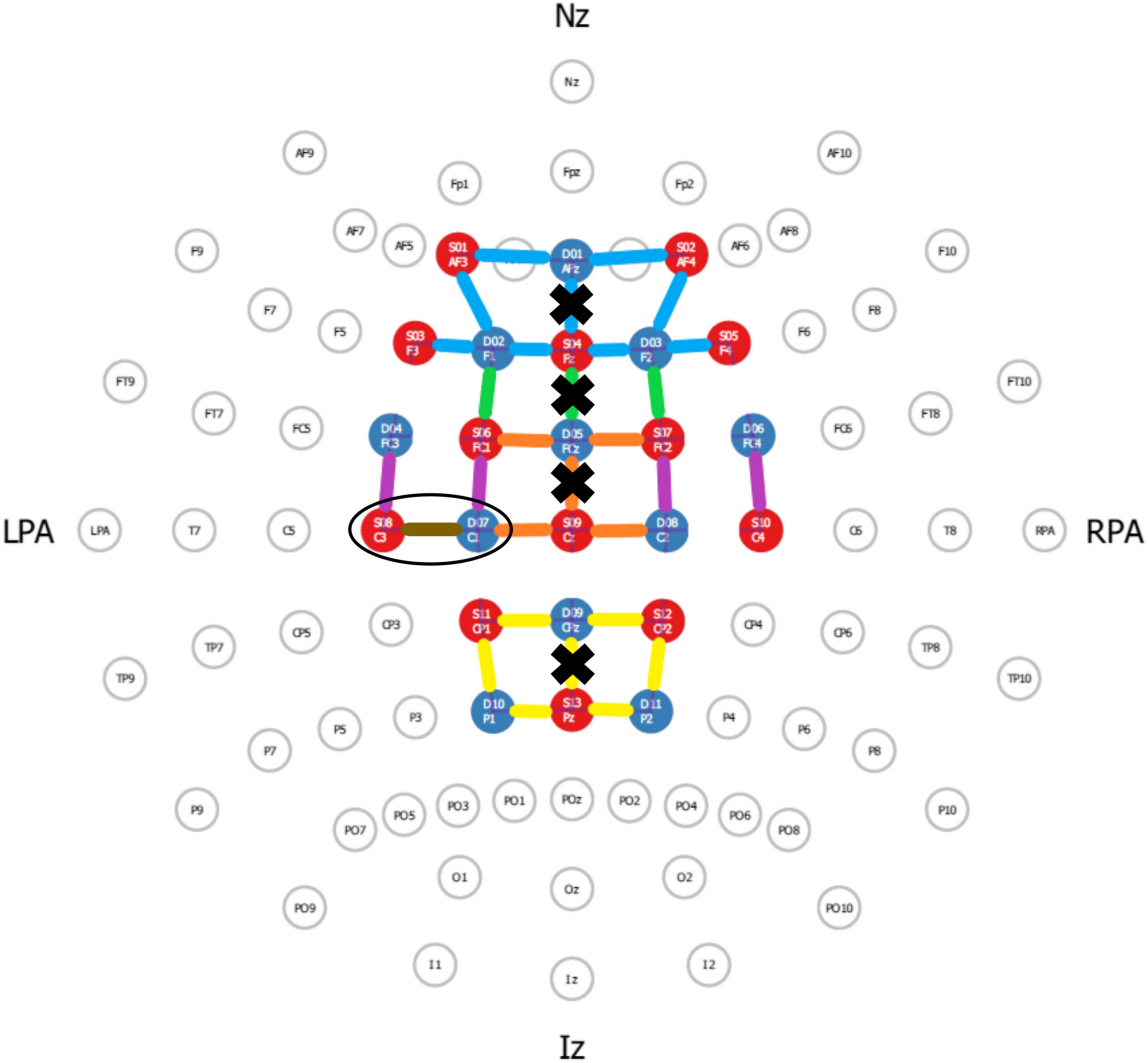
fNIRS lay-out with ROIs according to the fOLD toolbox for the postural task with a specificity level ≥50%. The PFC is represented by the blue colored channels, the FEF by the green channels, the SMA by the orange channels, the PMC by the purple channels, and the SSC by the yellow channels. The channels situated at the midline were excluded (indicated by black crosses). The C3-C1 channel (black circle; brown channel) was included as the task-specific hand motor channel for the finger tapping task, consistent with the EEG topography. Nz, naison; Iz, inion; LPA, left point auricular; and RPA, right point auricular.

Channels were averaged within the predefined ROIs. A channel was included if the spatial specificity, as determined with the fOLD toolbox,^34^ was at least 50% within a ROI^43^ (see Fig. 1). As the SMA and PMC ROIs were grouped within the fOLD output, the medial channels were defined as SMA and the lateral channels as PMC.^44^ The primary motor cortex (M1) was not included as ROI in this analysis, because spatial specificity did not exceed 50%. Midline-traversing channels were excluded, as cerebrospinal fluid running through the superior sagittal sinus could have interfered with the measurement of the underlying brain hemodynamics.^45^ In addition, the tapping task-specific hand motor channel C3-C1 was excluded from the ROIs specified for the postural task, as spatial specificity was lower than 50% (35% M1 and 35% PMC). Trial-averaged relative oxygenated hemoglobin (${\mathrm{HbO}}_{2}$ active trial - ${\mathrm{HbO}}_{2}$ rest trial (μmol/L)) was used as the primary outcome for each ROI. As secondary outcomes, HHb (μmol/L) and ${\mathrm{HbO}}_{2}$ (μmol/L) values for the left and right ROIs were investigated separately.

To a-posteriori check which channels were active during the postural or the finger-tapping task, signal changes in individual channels were assessed in a complementary analysis. A channel was classified as active when there was a significant average change during the task compared to rest in both ${\mathrm{HbO}}_{2}$ (positive change) and HHb levels (negative change) over the three time points, which was FDR-corrected for the number of channels.

### 2.4 Statistical Analysis

Statistical analyses were performed with SPSS (IBM SPSS Statistics, version 26). After checking the data distribution, test-retest differences in behavioral performance on both the postural and tapping task were investigated using a repeated measures analysis of variance (ANOVA) with time (test 1, 2, and 3) as within-subject factor. The assumption of sphericity was checked, and Greenhouse-Geisser correction used in case of violation. The same approach tested differences between time points for the fNIRS outcomes, and post-hoc tests were Bonferroni corrected for multiple comparisons. Next, single and average ICCs, as well as the corresponding confidence intervals (CIs), were calculated using a two-way mixed model with absolute agreement.^25^^,^^46^ Single measure ICCs are reported as the primary measure of test-retest reliability, as only one fNIRS measurement (consisting of seven trials) was performed during each test session.^25^^,^^46^ ICCs were obtained between test 1 and 2 and between test 1 and 3, investigating test-retest reliability on the same day and on consecutive days after cap removal, respectively. ICCs were interpreted as poor (ICC<0.40), fair (0.40≤ICC<0.60), good (0.60≤ICC<0.75), or excellent (0.75≤ICC<1.00).^25^ To allow for a meaningful interpretation, negative ICC values were replaced by zero.^25^ Additionally, the standard error of measurement (SEM) was calculated as

$$\text{SDpooled}*\sqrt{1-\mathrm{ICC}},$$ (2)

where SDpooled refers to the average standard deviation (SD) across measurements.^47^^,^^48^ Finally, Bland–Altman plots are presented to visualize the mean difference in relation to the average ${\mathrm{HbO}}_{2}$ levels of the two tests for each subject separately.^49^

## 3 Results

### 3.1 Participants’ Characteristics

Twenty-two healthy older adults were recruited for this study, as part of the control group for a larger RCT (clinicaltrial.gov ID: NCT04594148). Two were excluded due to not meeting the in-/exclusion criteria (one had a MoCA score<26 and one was aged below 65 years). Participant characteristics of the included 20 healthy older adults are shown in Table 1. They were all right-handed (self-reported).

**Table 1:** Participant characteristics.

	Dataset (N=20)
Gender (m/f)	10/10
Age (years)	71.00 (67.3 - 75.0)
Height (cm)	1.69 ± 0.1
Weight (kg)	71.17 ± 11.8
MiniBEST (0-28)	24.95 ± 1.9
FES-I (16-64)	19.00 (17.0, 23.5)
MoCA (0-30)	28.00 (26.3 - 29.8)

Note: Normally distributed data are displayed as mean ± SD, and not normally distributed data as median (first quartile - third quartile). MiniBEST, mini balance evaluation systems test; FES-I, Falls Efficacy Scale International; and MoCA, montreal cognitive assessment. MiniBEST, FES-I, and MoCA are presented for descriptive purposes only.

### 3.2 Behavioral Results

Even though the postural task was not standardized, weight-shifting performance was similar across the three test sessions, as no differences were found in speed [$F_{\left(1.53\right)}=0.35$, p=0.65, $\eta_{p}^{2}=0.02$; see Figure S3(a) in the Supplementary Material] and accuracy [$F_{\left(2\right)}=0.52$, p=0.60, $\eta_{p}^{2\;}=0.03$, see Figure S3(b) in the Supplementary Material]. Similar results were found for tapping performance [accuracy: $F_{\left(1.50\right)}=1.70$, p=0.21, $\eta_{p}^{2}=0.09$, see Figure S3(c) in the Supplementary Material; number of taps: $F_{\left(1.49\right)}=1.30$, p=0.28, $\eta_{p}^{2}=0.07$, see Figure S3(d) in the Supplementary Material]. Exploratory post-hoc tests with Bonferroni corrections for multiple testing also did not show any difference between tests (postural: corrected p-values>0.30; tapping: corrected p-values>0.07). It should be noted, however, that there was a trend towards a better accuracy for tapping in test 2 compared to test 1 (p=0.07).

### 3.3 fNIRS Results

**Fig. 2:**
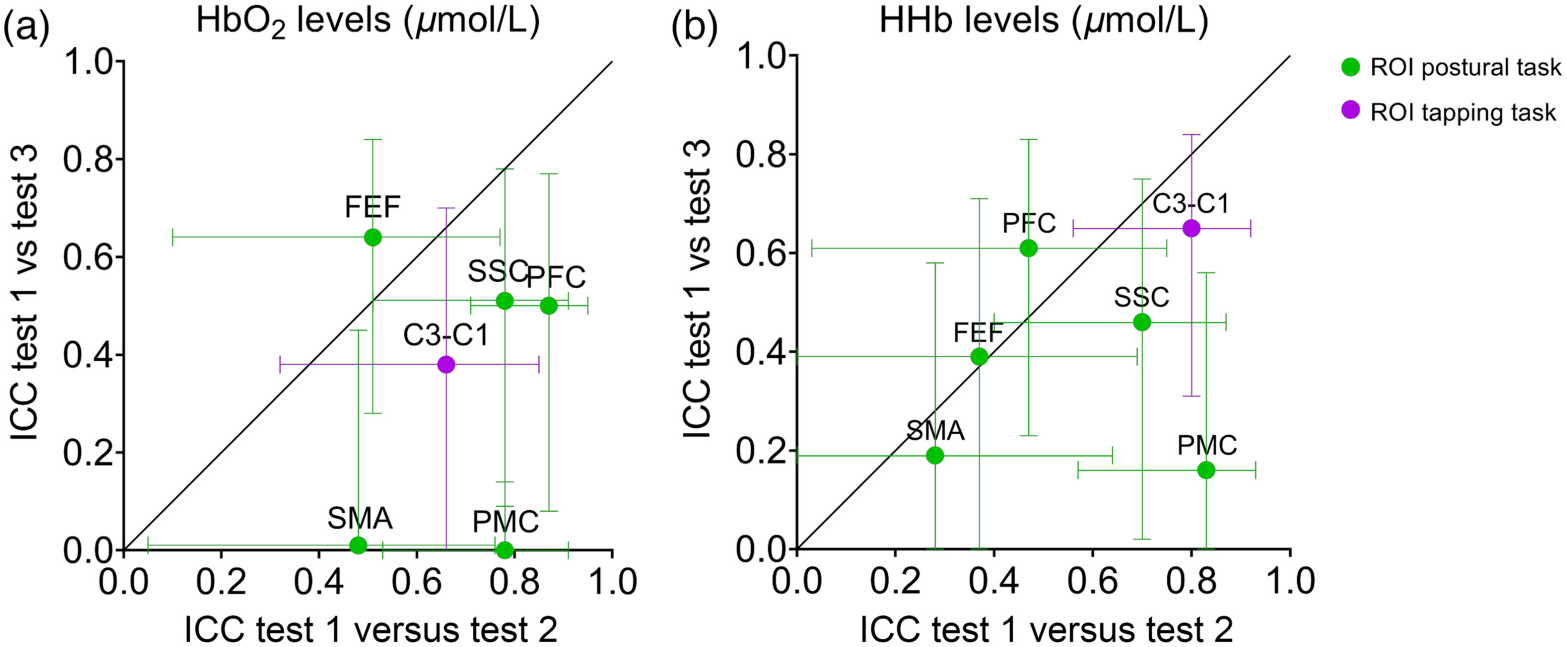
Intraclass correlations (ICC) and CIs of test 1 versus test 2 and test 1 versus test 3 for both (a) oxygenated (${\mathrm{HbO}}_{2}$) and (b) deoxygenated (HHb) hemoglobin. ROIs of the weight-shifting task are displayed with green dot (ICC) and line (CI) and the task-specific ROI of the right finger tapping task (channel C3-C1) are represented by the purple dot (ICC) and line (CI).

Figure 2 gives an overview of the ICCs and CIs between test 1-2 versus test 1-3 for both ${\mathrm{HbO}}_{2}$ and HHb levels. This illustrates that for both outcomes, ICCs were higher for test 1-2 compared to test 1-3 and this particularly in SMA and PMC. The below sections describe these results in more detail.

#### 3.3.1 ROI reliability during the postural task without cap repositioning

**Fig. 3:**
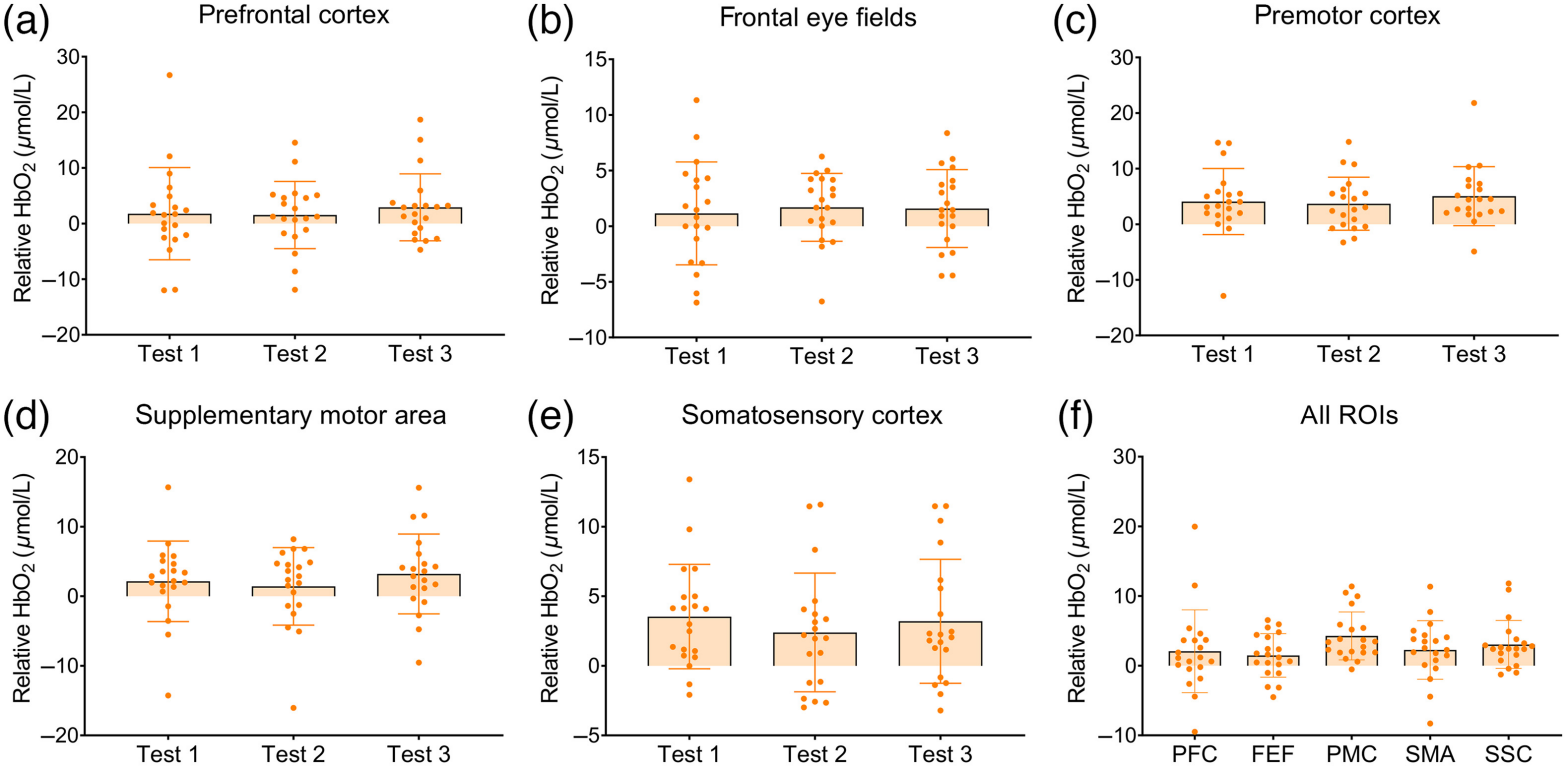
(a)–(e) Relative ${\mathrm{HbO}}_{2}$ levels (active trial - rest trial) for total (left plus right channels) ROIs at test 1, 2, and 3 for the postural task. Note that the scale on the x-axis differs between ROIs as to visualize the slightest differences. (f) Relative ${\mathrm{HbO}}_{2}$ levels for all total ROIs averaged over the three test moments. ROI, region of interest; PFC, prefrontal cortex; FEF, frontal eye fields; PMC, premotor cortex; SMA, supplementary motor area; and SSC, somatosensory cortex.

**Fig. 4:**
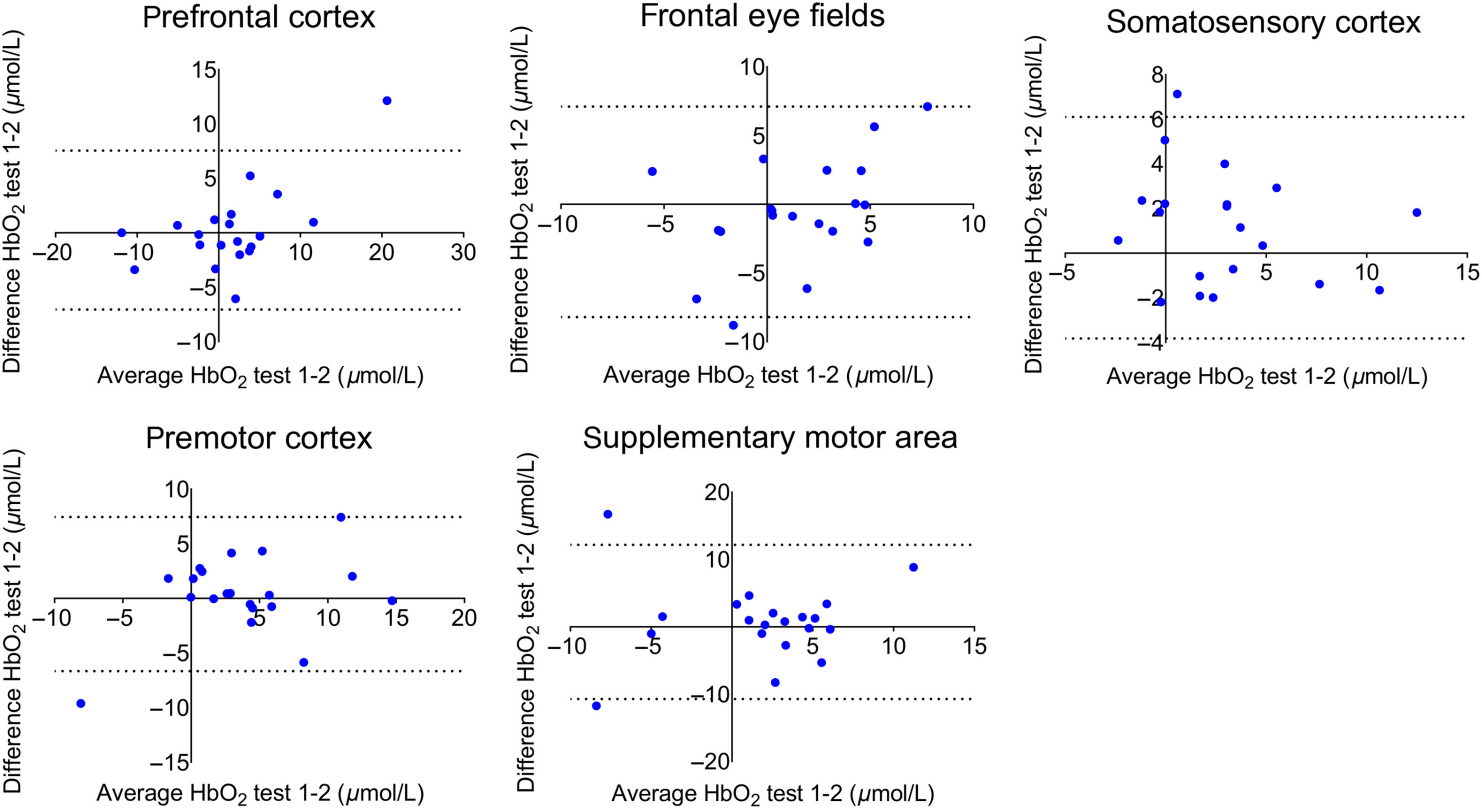
Bland–Altman plots for total (left plus right channels) ROIs during the postural task, visualizing the average ${\mathrm{HbO}}_{2}$ levels (x-axis) and the difference in ${\mathrm{HbO}}_{2}$ levels between test 1 and test 2 (y-axis). Dotted lines represent the 95% limits of agreement.

In the total ROIs (left plus right channels), no differences in ${\mathrm{HbO}}_{2}$ were found across time points [PFC: $F_{\left(1.41\right)}=0.62$, p=0.49; FEFs: $F_{\left(2\right)}=0.25$, p=0.78; SMA: $F_{\left(2\right)}=0.73$, p=0.49; PMC: $F_{\left(1.20\right)}=0.39$, p=0.58; SSC: $F_{\left(1.35\right)}=0.83$, p=0.40; see Figs. 3(a)–3(e)]. Exploratory post-hoc tests also did not show differences between the three tests (all corrected p-values>0.17). The ${\mathrm{HbO}}_{2}$ test-retest reliability, as captured by the ICC-values and CIs between test 1-2 was excellent for the PFC (ICC=0.87, CI=[0.71,0.95]), PMC (ICC=0.78, CI=[0.53,0.91]) and SSC (ICC=0.78, CI=[0.51,0.91]), and fair for the FEF (ICC=0.51, CI=[0.10,0.77]) and SMA (ICC=0.48, CI=[0.05,0.76]) (see Table 2). Table 2 further illustrates that the reliability for the ROIs per hemisphere showed lower values, but still within an acceptable range (PFC: ICC≥0.76; FEF: ICC≥0.49; SMA: ICC≥0.40; PMC: ICC≥0.74; SSC: ICC≥0.60). Bland–Altman plots are shown in Fig. 4. Points fell largely within the limits of agreement, were equally distributed around zero, and showed no bias, corroborating the findings above. The pattern, which was overall similar for the different ROIs, did show that one participant with extreme activation (PFC) or deactivation (PMC, SMA) values also had the least consistency between time points. As for the SEMs, values ranged between 1.89 and 4.09  μmol/L for the total ROIs and between 2.51 and 4.91  μmol/L for the ROIs per hemisphere.

**Table 2:** Test-retest reliability based on single ICCs for relative ${\mathrm{HbO}}_{2}$ and HHb levels during the postural task.

			Mean ± SD (μmol/L)			ICC (95% CI)		SEM (μmol/L)	
			Test 1	Test 2	Test 3	Test 1-2	Test 1-3	Test 1-2	Test 1-3
${\mathrm{HbO}}_{2}$	PFC	Total	1.78 ± 8.28	1.53 ± 6.04	2.92 ± 6.02	0.87 (0.71, 0.95)**	0.50 (0.08, 0.77)*	2.58	5.13
		Left	2.39 ± 10.47	1.12 ± 7.61	1.94 ± 6.58	0.77 (0.50, 0.90)**	0.66 (0.32, 0.85)**	4.44	5.08
		Right	1.17 ± 7.78	1.94 ± 6.48	3.90 ± 6.78	0.84 (0.64, 0.93)**	0.00 (0.00, 0.39)	2.89	7.30
	FEF	Total	1.15 ± 4.63	1.70 ± 3.05	1.58 ± 3.50	0.51 (0.10, 0.77)*	0.64 (0.28, 0.84)**	2.74	2.47
		Teft	1.40 ± 4.47	1.77 ± 4.16	0.72 ± 4.65	0.49 (0.06, 0.76)*	0.31 (0.00, 0.65)	3.10	3.80
		Right	0.90 ± 5.98	1.63 ± 4.13	2.45 ± 4.02	0.59 (0.21, 0.81)**	0.48 (0.08, 0.76)*	3.29	3.66
	SMA	Total	2.15 ± 5.79	1.42 ± 5.57	3.20 ± 5.74	0.48 (0.05, 0.76)*	0.01 (0.00, 0.45)	4.09	5.73
		Left	1.82 ± 6.55	1.73 ± 4.90	3.43 ± 5.67	0.49 (0.07, 0.77)*	0.22 (0.00, 0.60)	4.11	5.40
		Right	2.48 ± 5.97	1.11 ± 6.72	2.98 ± 6.91	0.40 (0.00, 0.71)*	0.00 (0.00, 0.43)	4.91	6.45
	PMC	Total	4.08 ± 5.95	3.68 ± 4.77	5.05 ± 5.30	0.78 (0.53, 0.91)**	0.00 (0.00, 0.14)	2.51	5.63
		Left	4.16 ± 7.29	3.62 ± 5.73	5.11 ± 6.35	0.74 (0.45, 0.89)**	0.00 (0.00, 0.09)	3.36	6.83
		Right	4.00 ± 5.69	3.75 ± 5.29	5.00 ± 5.53	0.74 (0.46, 0.89)**	0.01 (0.00, 0.45)	2.78	5.60
	SSC	Total	3.54 ± 3.75	2.40 ± 4.26	3.20 ± 4.45	0.78 (0.51, 0.91)**	0.51 (0.09, 0.78)*	1.89	2.87
		Left	2.98 ± 4.07	2.37 ± 3.85	3.29 ± 5.46	0.60 (0.23, 0.82)**	0.06 (0.00, 0.49)	2.51	4.66
		Right	4.09 ± 5.41	2.43 ± 5.27	3.12 ± 5.34	0.73 (0.43, 0.89)**	0.65 (0.31, 0.85)**	2.76	3.18
HHb	PFC	Total	0.97 ± 3.24	0.56 ± 2.98	0.88 ± 3.31	0.47 (0.03,0.75)*	0.61 (0.23,0.83)**	2.28	2.05
		Left	0.64 ± 3.93	7.54 ± 4.36	1.05 ± 3.60	0.30 (0.00, 0.66)	0.37 (0.00, 0.70)	3.47	2.98
		Right	1.31 ± 3.46	0.37 ± 2.24	0.72 ± 3.28	0.63 (0.28, 0.83)**	0.72 (0.43, 0.88)**	1.78	1.78
	FEF	Total	0.57 ± 3.21	0.03 ± 1.55	0.25 ± 1.74	0.37 (0.00, 0.69)	0.39 (0.00, 0.71)*	2.00	2.02
		Left	−0.06 ± 1.48	0.22 ± 1.65	0.27 ± 2.19	0.29 (0.00, 0.65)	0.34 (0.00, 0.67)	1.32	1.52
		Right	1.20 ± 5.64	−0.15 ± 2.14	0.24 ± 1.91	0.44 (0.03, 0.73)*	0.28 (0.00, 0.64)	3.18	3.56
	SMA	Total	−0.31 ± 3.78	−0.74 ± 2.52	-0.10 ± 2.28	0.28 (0.00, 0.64)	0.19 (0.00, 0.58)	2.72	2.81
		Left	−0.53 ± 4.29	−0.62 ± 2.69	−0.12 ± 2.80	0.25 (0.00, 0.62)	0.25 (0.00, 0.62)	3.09	3.14
		Right	−0.08 ± 3.65	−0.86 ± 2.59	−0.09 ± 2.43	0.31 (0.00, 0.65)	0.14 (0.00, 0.55)	2.63	2.88
	PMC	Total	0.26 ± 2.26	−0.44 ± 2.52	0.14 ± 2.28	0.83 (0.57, 0.93)**	0.16 (0.00, 0.56)	0.99	2.08
		Left	0.14 ± 3.23	−0.26 ± 3.52	0.27 ± 2.63	0.86 (0.69, 0.94)**	0.00 (0.00, 0.45)	1.25	2.94
		Right	0.37 ± 1.86	−0.61 ± 2.18	0.01 ± 2.60	0.69 (0.25, 0.88)**	0.41 (0.00, 0.72)*	1.12	1.74
	SSC	Total	−1.00 ± 1.22	−1.28 ± 1.43	−1.08 ± 1.14	0.70 (0.40, 0.87)**	0.46 (0.02, 0.75)*	0.72	0.87
		Left	−0.98 ± 1.66	−1.22 ± 1.66	−1.13 ± 1.56	0.80 (0.58, 0.92)**	0.41 (0.00, 0.72)*	0.74	1.24
		Right	−1.01 ± 1.03	−1.34 ± 1.53	−1.03 ± 1.18	0.55 (0.17, 0.79)**	0.37 (0.00, 0.70)	0.88	0.88

Note: ${\mathrm{HbO}}_{2}$, oxygenated hemoglobin; HHb, deoxygenated hemoglobin; ROI, region of interest; SD, standard deviation; ICC, intraclass correlation coefficient; SEM, standard error of measurement; PFC, prefrontal cortex; FEF, frontal eye fields; SMA, supplementary motor area; PMC, premotor cortex; and SSC, somatosensory cortex.

* Significant at α<0.05.

** Significant at α<0.01.

Overall, HHb test-retest reliability was lower compared to ${\mathrm{HbO}}_{2}$ (ICC−0.15 on average for total ROIs), with some exceptions (see Table 2 and Fig. 2). Averaged ICCs, representing the test-retest reliability of the averaged test outcomes^50^ showed higher values [see Table S2 in the Supplementary Material (${\mathrm{HbO}}_{2}$) and Table S3 in the Supplementary Material (HHb)]. Additionally, individual channel-based analysis revealed that 13 out of 32 channels were activated during the postural weight-shifting task [see Figure S4(a) in the Supplementary Material], including those in the SSC, SMA, and PMC.

#### 3.3.2 ROI reliability during the postural task with cap repositioning

**Fig. 5:**
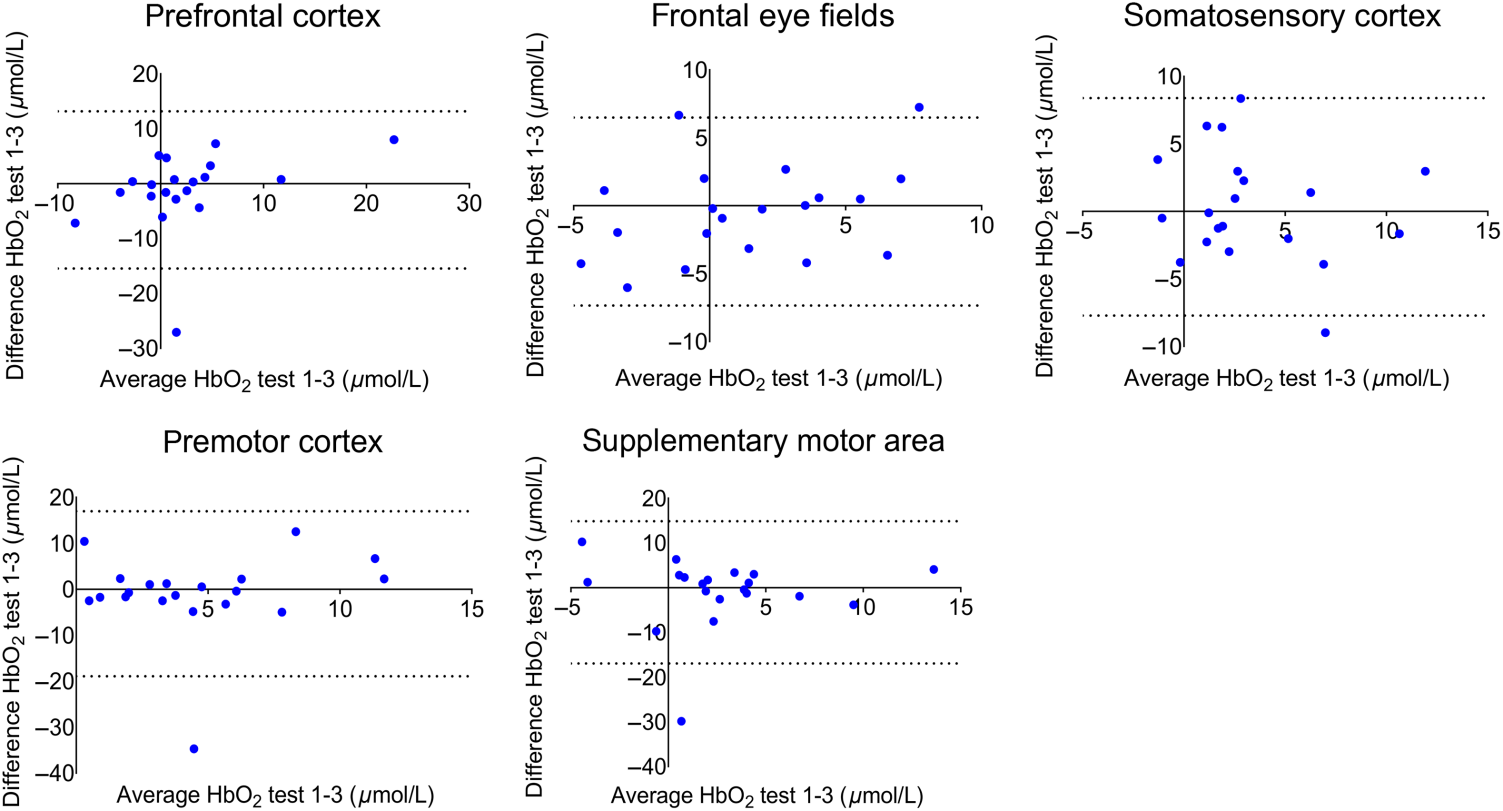
Bland–Altman plots for total (left plus right channels) ROIs during the postural task, visualizing the average ${\mathrm{HbO}}_{2}$ levels (x-axis) and the difference in ${\mathrm{HbO}}_{2}$ levels between test 1 and test 3 (y-axis). Dotted lines represent the 95% limits of agreement.

Table 2 also illustrates that the repositioning of the cap between test 1 and 3 led to less stable test-retest values than between test 1 and 2. Reliability values were lower for the PFC (ICC=0.50, CI=[0.08,0.77]) and SSC (ICC=0.51, CI=[0.09,0.78]), but remained fair. Reliability became poor for the SMA (ICC=0.01, CI=[0.00,0.45]) and the PMC (ICC=0.00, CI=[0.00,0.14]). However, reliability was good for the FEF (ICC=0.64, CI=[0.28,0.84]). Table 2 also shows that reliability for the ROIs per hemisphere was similar for the motor areas (SMA: ICC≤0.22; PMC: ICC≤0.01), and lower for the FEF (ICC≤0.48). Interestingly, ICC-values were higher for the left PFC (ICC=0.66, [0.32, 0.85]) and right SSC (ICC=0.65, [0.31, 0.85]), but lower for the right PFC (ICC=0.00, CI=[0.00,0.39]) and left SSC (ICC=0.06, [0.00, 0.49]). Bland–Altman plots of test 1 and 3 largely confirm the above described results (see Fig. 5). Limits of agreement increased for total ROIs compared to the test 1-2 comparison, especially for the PMC and SMA, but not for the FEF. The same participant with extreme (de)activation as previously mentioned, also showed the least consistency between tests 1 and 3 (PFC, PMC, SMA). SEM scores were also larger between test 1 and 3 (range: 2.47−5.73  μmol/L) than between test 1 and 2 (range: 2.51−4.09  μmol/L), with the FEF as the only exception (change in SEM: −0.27  μmol/L). SEMs ranged between 3.18 and 7.30  μmol/L for the ROIs per hemisphere.

The overall HHb test-retest reliability was comparable to ${\mathrm{HbO}}_{2}$ (ICC−0.03 on average for total ROIs), though less stable between test 1 and 3 than between test 1 and 2 (see Table 2 and Fig. 2). Averaged ICCs showed higher values for both ${\mathrm{HbO}}_{2}$ (see Table S2 in the Supplementary Material) and HHb (see Table S3 in the Supplementary Material).

#### 3.3.3 Task-specific fNIRS reliability during finger tapping

**Fig. 6:**
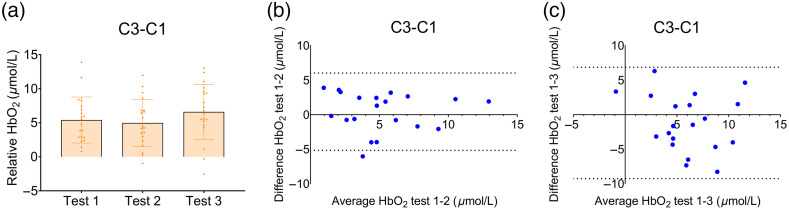
(a) Relative ${\mathrm{HbO}}_{2}$ levels (active trial - rest trial) at test 1, 2, and 3 for the hand motor channel C3-C1 during the finger tapping task; (b) Bland–Altman plot for channel C3-C1, visualizing the average ${\mathrm{HbO}}_{2}$ levels and the difference between ${\mathrm{HbO}}_{2}$ levels at test 1 and 2, and (c) test 2 and 3.

For the task-specific motor channel representing the right hand (C3-C1), no differences in relative ${\mathrm{HbO}}_{2}$ levels were found across time points during the tapping task [$F_{\left(1.53\right)}=1.75$, p=0.20; see Fig. 6(a)]. Exploratory post-hoc tests also did not show differences between test moments (all corrected p-values>0.46), even though a trend towards improved tapping accuracy was seen from test 1 to test 2. Interestingly, however, participants who showed better accuracy (lower tapping error) were as stable in their fNIRS outcomes, as assessed with Pearson’s correlations (${\mathrm{HbO}}_{2}$: r=0.30, p=0.20; HHb: r=−0.33, p=0.15; see Figure S5 in the Supplementary Material). The ICC-values were good between tests 1-2 (ICC=0.66, CI=[0.31,0.84]), but became poor between tests 1-3 (ICC=0.38, CI=[0.00,0.70]) (see Table 3). Bland–Altman plots largely confirm these results [see Figs. 6(b) and 6(c)]. Points fell between the limits of agreement, which covered a wider range and were more spread out between test 1 and 3 than between test 1 and 2. In agreement, the SEM score was 2.00  μmol/L between test 1 and 2, and 2.93  μmol/L between test 1 and 3.

**Table 3:** Test-retest reliability based on single ICCs for relative ${\mathrm{HbO}}_{2}$ and HHb levels during the finger tapping task.

	Mean ± SD (μmol/L)			ICC (95% CI)		SEM (μmol/L)	
	Test 1	Test 2	Test 3	Test 1-2	Test 1-3	Test 1-2	Test 1-3
HbO	5.40 ± 3.38	4.97 ± 3.46	6.60 ± 4.04	0.66 (0.32-0.85)**	0.38 (0.00-0.70)*	2.00	2.93
HHb	−1.73 ± 1.46	−1.58 ± 1.28	−2.11 ± 2.05	0.80 (0.56-0.92)**	0.65 (0.31-0.84)**	0.62	1.06

Note: Values are determined for the task-specific motor channel C3-C1. ${\mathrm{HbO}}_{2}$, oxygenated hemoglobin; HHb, deoxygenated hemoglobin; SD, standard deviation; ICC, intraclass correlation coefficient; and SEM = standard error of measurement.

* Significant at α<0.05.

** Significant at α<0.01.

HHb test-retest reliability, as well as the ${\mathrm{HbO}}_{2}$ and HHb averaged ICCs (see Table S4 in the Supplementary Material), were better than the single ICCs for ${\mathrm{HbO}}_{2}$, ranging from fair to excellent. Additionally, individual channel-based analysis revealed that five channels of the left hemisphere were active during the right finger-tapping task [see Fig. S4(b) in the Supplementary Material], including the C3-C1 channel, which confirmed the anatomical location of the right-hand motor region.

## 4 Discussion

### 4.1 Main Study Findings

This is the first study to investigate fNIRS test-retest reliability of a postural weight-shifting and finger tapping control task in healthy older adults. When performing two measurements with an in-between resting period of 30 min and the cap remained in place, results showed fair (FEF, SMA) to excellent (PFC, PMC, and SSC) reliability during a postural task in five predefined ROIs and good reliability during tapping in one task-specific motor channel. Contrary to our hypothesis, these results are similar to published results in young healthy adults. However, as expected when testing on separate days after removal of the cap, reliability was reduced, and reliability became fair (PFC and SSC) to good (FEF) for the non-motor areas, and poor for the motor areas (SMA, PMC, and C3-C1 hand motor channel). These results are largely in line with previous studies, as detailed in Table 4, with the exception that we found poor reliability in the motor areas.

**Table 4:** Comparison between the current work and prior studies on fNIRS test-retest reliability in various motor tasks.

Study	Participants	Task	Brain region	Test interval^a^	Cap replacement^b^	Reliability (ICC)^c^
Current study	20 older adults 67.3 ± 75.0 years	VR weight-shifting right finger-tapping	PFC FEF SMA PMC SSC right hand motor area	30 min, 24 hrs	Markings on head Cz, CP2, FC1	${\mathrm{HbO}}_{2}=0.48$ to 0.87 (30 min), ${\mathrm{HbO}}_{2}=0.00$ to 0.64 (24 hr), HHb = 0.28 to 0.83 (30 min), HHb = 0.16 to 0.65 (24 hr)
1. Wyser et al.^20^	15 young adults 27.0 ± 4.6 years	Self-paced, isometric, hand grasping practiced with metronome (∼1 Hz) right & left hand	Primary motor cortex bilateral (task-specific)	5.5 ± 3.1 days	TMS localization	${\mathrm{HbO}}_{2}=0.62$ to 0.79 HHb = 0.73 to 0.81
2. Bae et al.^21^	5 young adults mean: 21.8 years range: 21-23 years	Robotic passive hand movement right hand	Primary sensorimotor cortex left	10 sessions 1, 3, 7, 23 days, 15 min, 6 hr	3D digitizer	${\mathrm{HbO}}_{2}=0.002$ (overall)
3. Broscheid et al.^26^	20 healthy adults 42.2 ± 9.8 years 20 MS-patients 41.0 ± 12.0 years	Self-paced walking back and forth 12 m for 6 min	PFC bilateral	24 hrs	Not mentioned	${\mathrm{HbO}}_{2}=0.39$ to 0.74 (healthy) HHb = 0.39 to 0.63 (healthy) ${\mathrm{HbO}}_{2}=0.00$ to 0.39 (MS) HHb = 0.20 to 0.56 (MS)
4. Stuart et al.^27^	25 young adults 32.3 ± 7.5 years	2 min walking 2 min turning	PFC bilateral	5 to 10 min	3D digitizer	${\mathrm{HbO}}_{2}=0.67$ (walking) ${\mathrm{HbO}}_{2}=0.71$ (turning)
5. Dravida et al.^22^	14 young adults 26.9 ± 9.5 years	cued digit manipulation tasks right hand, four different tasks	motor cortex bilateral (task-specific)	1 day	3D digitizer	${\mathrm{HbO}}_{2}=0.51$ to 0.83 HHb = 0.38 to 0.67
6. Plichta et al.^24^	12 young adults 29.1 ± 6.0 years	Visually cued index finger-tapping ∼2.5 Hz, left and right	Motor cortex bilateral	3 weeks	Not mentioned	${\mathrm{HbO}}_{2}=0.70$ to 0.74 HHb = 0.67 to 0.77
7. Bhambhani et al.^23^	13 healthy adults 31.5 ± 4.5 years 25 TBI-patients 31.6 ± 9.8 years	Maximal rhythmic handgrip exercises right hand	PFC left	24 to 48 hrs	Not mentioned	${\mathrm{HbO}}_{2}=0.83$ (healthy) ${\mathrm{HbO}}_{2}=0.70$ (TBI)

Note: ICC, intraclass correlation coefficient; ${\mathrm{HbO}}_{2}$, oxygenated hemoglobin; HHb, deoxygenated hemoglobin; and TMS, transcranial magnetic stimulation.

a Cap removed between tests in all studies;

b method used for ensuring similar cap (re)placement at the different tests;

c single-measure; ICC, intraclass correlation coefficient

Behaviorally, performance on the postural and tapping task did not show significant improvements over time, suggesting that no test-induced learning effects were present. However, a trend towards more accurate tapping was shown from test 1 to test 2, despite providing auditory pacing. Interestingly, this improvement in behavioral accuracy was not associated with a change in fNIRS outcomes. Previous investigations on fNIRS test-retest reliability did not report on the stability of the behavioral correlates.^20^^–^^24^^,^^26^^,^^27^ Four of these studies (study 1, 2, 5, and 6 in Table 4) standardized performance to some extent to prevent a learning effect, for instance by using cues,^22^ robotic passive hand movements,^21^ practicing the task with a metronome,^20^ or using a very small time-window (1200 ms).^24^ Furthermore, one study (study 3 in Table 4) checked for possible differences in exhaustion via a Borg scale, showing no differences between test moments.^26^

### 4.2 fNIRS Reliability

#### 4.2.1 Reliability during postural control without cap removal

Test-retest analysis of five predefined and commonly applied ROIs during the postural task revealed an overall fair to excellent reliability in ${\mathrm{HbO}}_{2}$ levels when repeating two tests on the same day. This suggests that, when not removing the fNIRS cap and optodes, the fNIRS signal is fairly reliable over time for the FEF and SMA (ICC≥0.48) and highly reliable for the PFC, PMC, and SSC (ICC≥0.78). Similar results were found for the left and right hemispheres separately. To date, only one other study specifically investigated fNIRS test-retest reliability when testing twice on the same day (study 4 in Table 4),^27^ although with cap removal. These authors studied only the PFC in young adults, resulting in a good reliability during both straight walking and turning (ICC≥0.67). The higher reliability found in the current study for the postural task may be explained by not removing the cap, the lack of activity in this area, the shorter duration of signal extraction (20 sec versus 2 min) and higher number of trials (7 versus 1) in which the task was performed.^5^ Pilot testing prior to data collection also showed a 13% true positive rate increase by adding two trials (67% at five trials and 80% at seven trials). Additionally, other task-related aspects, such as heel strikes during gait, may induce movement artifacts, which were absent during the present postural task. As Stuart et al.^27^ did not report on HHb, the overall lower reliability compared to ${\mathrm{HbO}}_{2}$ found in this study cannot directly be compared.

#### 4.2.2 Reliability during postural control with cap removal

An important finding of the present study is that when measuring fNIRS ${\mathrm{HbO}}_{2}$ levels on different days after cap removal and repositioning, reliability deteriorated. Interestingly, non-motor cortical areas seemed less affected by cap removal, indicated by fair to good reliability in the PFC, FEF, and SSC (ICC≥0.50), which is comparable to the reliability found during walking^26^ and handgrip exercises^23^ (study 3 and 7 in Table 4). On the other hand, poor reliability was revealed in the motor areas (ICC≤0.01), in agreement with a reliability study during passive hand movements^21^ (study 2 in Table 4), possibly driven by the lack of active movement. The finding that the non-motor areas proved to be more stable across consecutive days could be due the fact that this virtual reality-based postural task required strong non-motor involvement. The relatively poor reliability found for the motor areas during the postural task (ICC≤0.01) is in contrast with previous research on hand grasping and finger tapping, which showed fair to excellent reliability after cap removal (ICC≥0.50) (study 1, 5, and 6 in Table 4).^20^^,^^22^^,^^24^ Besides cap repositioning, this finding may alternatively be explained by the fact that the postural task required whole body movements, resulting in wider and more variable cortical recruitment. The single channel-based analysis underscored this statement, as no clear activation pattern was found for the PMC and SMA. In addition, fNIRS signals pertaining to the lower limbs are more prone to systematic artifacts^29^ and likely more difficult to capture as the motor representation of the lower extremities is located deeply within the interhemispheric fissure,^37^ which could result in more inconsistent fNIRS measurements. Note that, similar to the present findings, HHb reliability after cap removal was overall comparable to ${\mathrm{HbO}}_{2}$ findings in healthy adults (see Table 4).

#### 4.2.3 Task-specific fNIRS reliability during finger tapping

For finger tapping, reliability of the C3-C1 hand motor channel was good when assessed on the same day (ICC=0.66). This supports the notion that fNIRS is a sensitive neuroimaging tool for capturing ${\mathrm{HbO}}_{2}$ changes during a specific motor tasks requiring localized cortical activation.^29^ It should be noted that there was a trend towards better behavioral tapping accuracy from test 1 to test 2 (p=0.07). However, when exploring this result, we did not find differences in the ${\mathrm{HbO}}_{2}$ levels or that the participants with improved accuracies had better reliability. Similar to the motor cortical ROIs of the postural task, reliability during tapping became poor when assessed on two consecutive days (ICC=0.38), though the decline was less steep (−0.47 (SMA) and −0.78 (PMC) for the postural task and −0.28 (C3-C1) for the tapping task). This highlights that using fNIRS on 2 consecutive days results in suboptimal reproducibility, despite efforts to standardize cap replacement. In contrast, previous studies investigating task-specific motor areas showed fair to excellent ICC-values (≥0.51) during digit manipulation^22^ and hand grasping^20^ when assessed on different days (study 1 and 5 in Table 4). These results, however, were found in young adults, who might show higher signal to noise ratios and therefore higher reliability.^28^ It could also be that the localization method used in these studies, TMS localization^20^ and registration of 3D-coordinates,^22^ provided a more accurate foundation for cap replacement after removal.

#### 4.2.4 Suggestions for improving fNIRS reliability

First, other methods of cap standardization could be explored, such as using a 3D-digitizer, a neuronavigation system^51^ or structural MRI scans for co-registration.^52^ Interestingly, we found that averaged ICCs were generally higher than single ICCs.^25^^,^^46^ Especially for study protocols that involve cap removal, multiple fNIRS blocks within one testing session, and the use of averaged values of ${\mathrm{HbO}}_{2}$ and HHb levels for between-test comparisons may be required. Second, future studies should investigate whether shortening of trial duration while increasing the number of trials within a block design, could lead to enhanced stability of fNIRS outcomes, as pilot testing revealed increased positive rates using this procedure. Third, behavioral tasks should be standardized as much as possible and any learning effects counteracted, as was done in the present study. Measuring on the same time of day and including large sample sizes is also recommended, especially when using a complex (motor) task. Finally, person-specific variables, such as vigilance, motivation, and effort, could additionally be assessed, as they can possibly affect reliability.

### 4.3 Strengths and Limitations

Despite a relatively small sample size and the inherently variable nature of fNIRS signals, we found an overall acceptable test-retest reliability of brain oxygenated hemoglobin levels as measured in five commonly used ROIs, as well as in a task-specific motor channel. Although this is the first study investigating test-retest reliability in older adults, future research should investigate whether the findings generalize to patient populations as well. Unlike previous studies, we examined the behavioral test-retest results to take any possible learning effects into account. Moreover, short-separation channels were used to correct for physiological noise in the fNIRS signal.^35^ A limitation of this study is that we used the same differential path length factor for all participants. It has been shown that age influences this factor during the conversion of optical density into ${\mathrm{HbO}}_{2}$, though it is not known for adults older than 50 years of age.^5^ Finally, fNIRS measurements are associated with high between-subject variance, as are the outcomes of other neuroimaging techniques, on which the ICC-value is dependent.^25^ Moreover, the range of CIs accompanying ICCs varied. Therefore, future studies in larger samples are needed to verify fNIRS test-retest reliability in healthy older adults and other study populations.

## 5 Conclusions

The present results indicate that repeated fNIRS measurements have fair to excellent reliability in healthy older adults during motor tasks, though reliability became poorer when measuring on multiple days after repositioning the cap.

## Code, Data, and Materials Availability

Study data and code are not publicly available due to privacy and ethical reasons. Data and code can be made available upon reasonable request to the corresponding author. Supplementary materials are provided online.
