# Supplementary material for: Test-retest reliability of functional near-infrared spectroscopy during a finger-tapping and postural task in healthy older adults
Source: Neurophotonics. 2023 May 26;10(2):025010. doi: 10.1117/1.NPh.10.2.025010 (PMC10218660; doi:10.1117/1.NPh.10.2.025010)
Supplement: Supplementary file 1 [file NPh_010_025010_SD001.pdf]

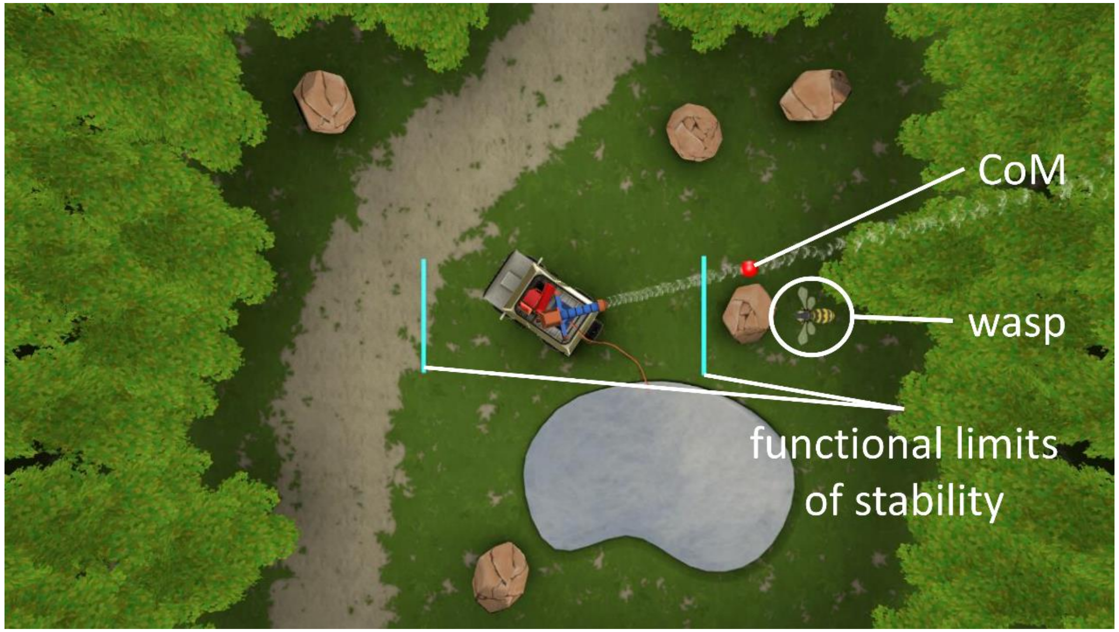

Fig. S1. Visualization of the postural task, which consisted of a weight-shifting wasp game. The turquoise lines indicate 80% of the individual's lateral limits of stability. The red dot displays the center of mass (CoM). By moving the CoM laterally beyond the turquoise lines, the water jet was activated and participants were instructed to try to hit as many wasps as possible appearing on the left or right side of the screen. Figure modified from de Rond et al. (2021)[1].

1. de Rond, V., Orcioli-Silva, D., Dijkstra, B. W., Orban de Xivry, J. J., Pantall, A., & Nieuwboer, A. (2021). Compromised Brain Activity With Age During a Game-Like Dynamic Balance Task: Single- vs. Dual-Task Performance. *Frontiers in Aging Neuroscience*, *13*(July), 1–13. https://doi.org/10.3389/fnagi.2021.657308

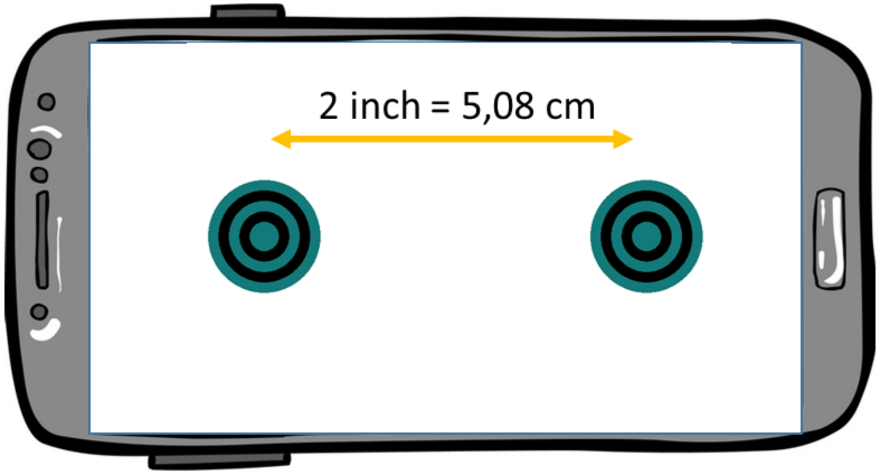

Fig. S2. Visualization of the finger tapping task embedded within the cloudUPDRS application on a smartphone. Two targets with a diameter of 0.6 inch were positioned at a distance of 2 inch. Figure modified from Broeder et al. (submitted)[2] with author permission.

2. Broeder, S., Roussos, G., De Vleeschhauwer, J., D'Cruz, N., Orban de Xivry, J.-J., & Nieuwboer, A. The feasibility and validity of a smartphone-based tapping task as a marker of medication response in Parkinson's disease. *In submission*, 2023.

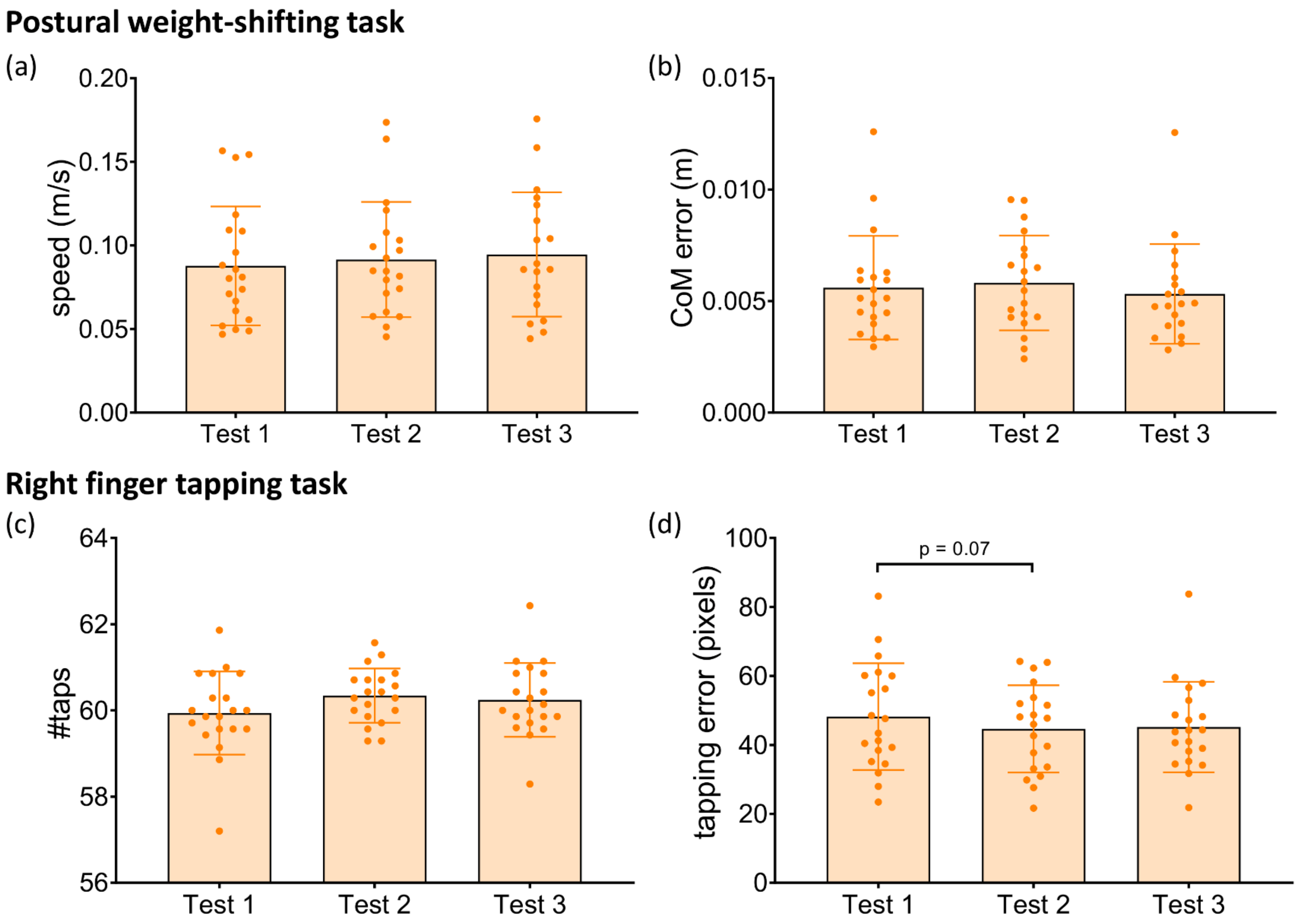

Fig. S3. Comparing behavioral outcomes of test 1, 2 and 3. (a) Weight-shifting speed and (n) Center of Mass (CoM) error (accuracy) during the postural weight-shifting task; and (c) the number of taps, and (d) tapping error (accuracy) during the finger tapping task.

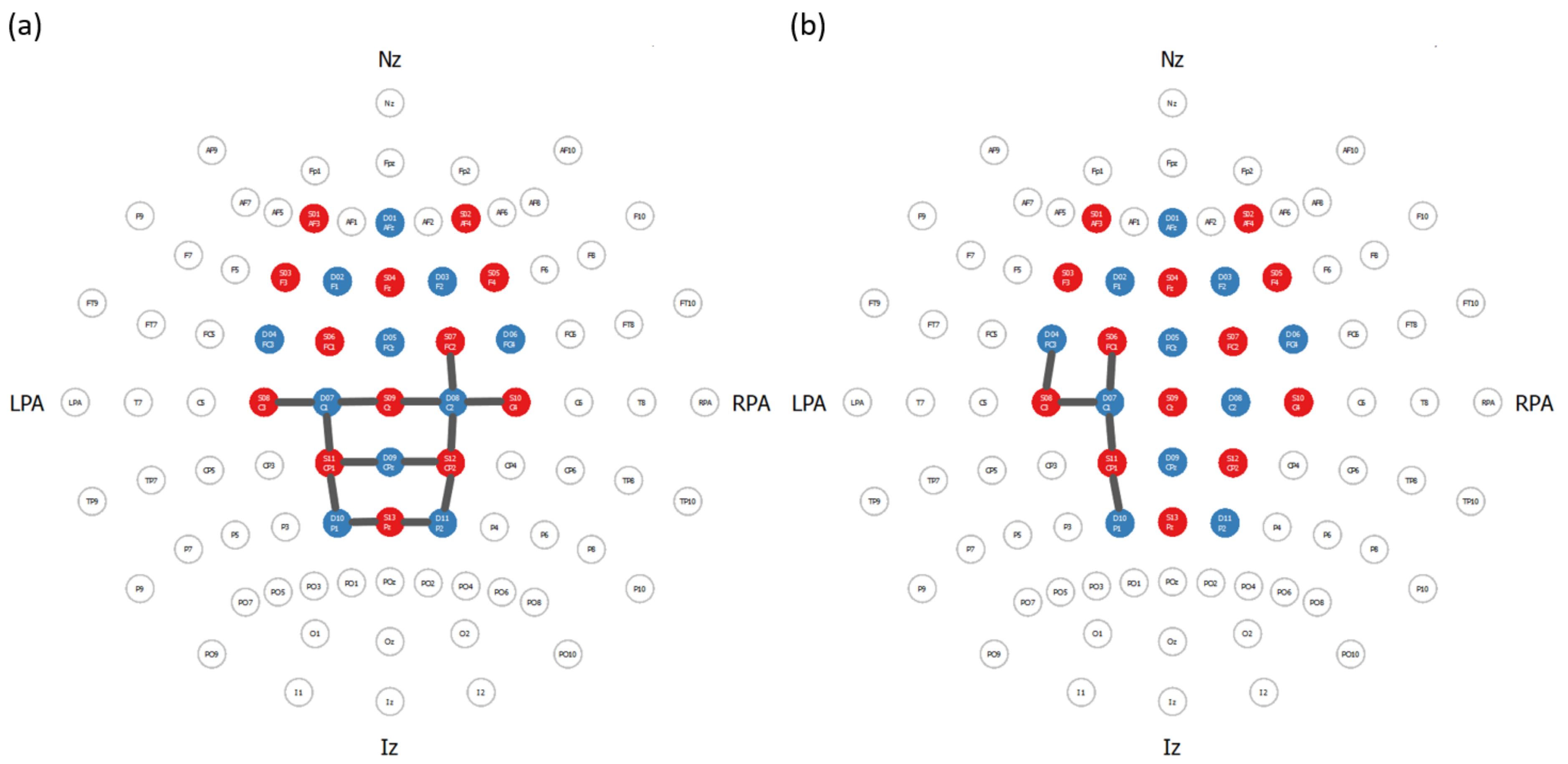

Fig. S4. fNIRS lay-out with active channels for the (a) postural and (b) right finger-tapping task. The channels situated at the midline were excluded from the analysis. Nz = naison. Iz = inion. LPA = left point auricular. RPA = right point auricular.

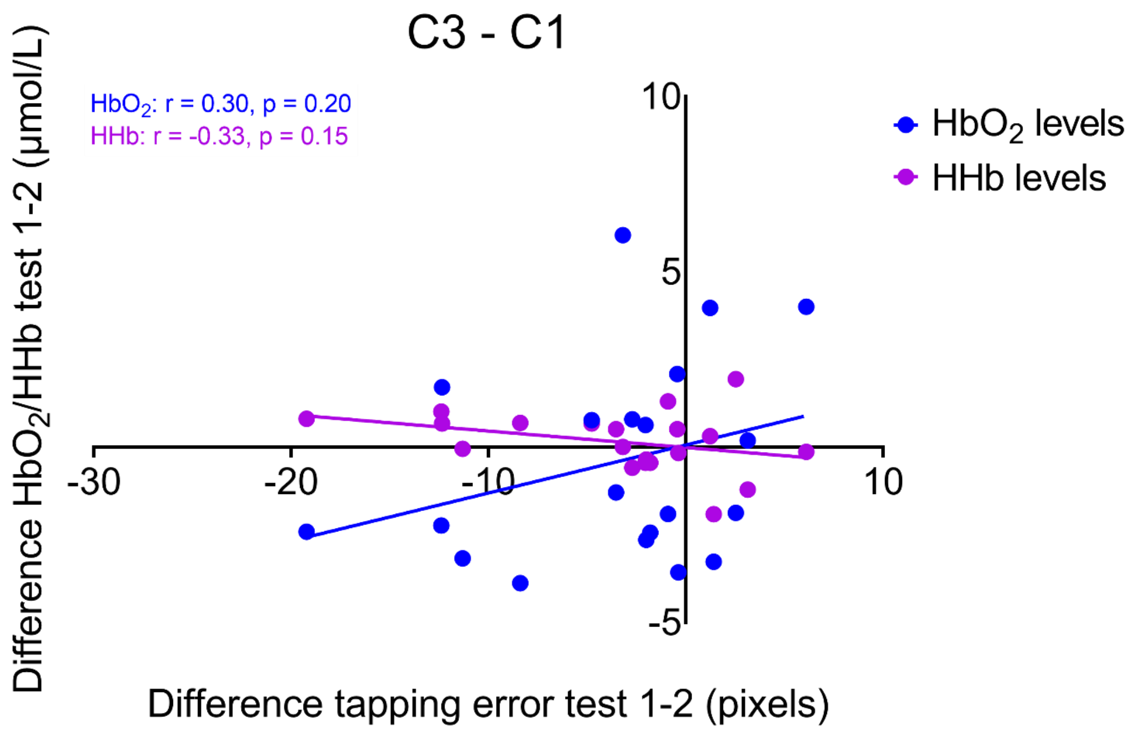

Fig. S5. Visualization of the difference in finger tapping error (accuracy; x-axis) and the difference in HbO_2 (blue dots) and HHb levels (purple dots) for the hand motor channel C3-C1 (y-axis). A lower difference in tapping error represents better accuracy at test 2 compared to test 1.

Table S1. Regions of interest (ROI) specification for the postural task

Source,	Optodes	Brodmann	Anatomic	Orientation	Specificity
1, 1	AF3, Afz	9, 10, 46	PFC	left	98.36
1, 2	AF3, F1	9, 10, 46	PFC	left	97.37
2, 1	AF4, Afz	9, 10, 46	PFC	right	97.42
2, 3	AF4, F2	9, 10, 46	PFC	right	96.35
3, 2	F3, F1	9, 46	PFC	left	91.46
4, 2	Fz, F1	9	PFC	left	63.16
4, 3	Fz, F2	9	PFC	right	68.93
5, 3	F4, F2	9, 46	PFC	right	90.77
6, 2	FC1, F1	8	FEF	left	63.72
7, 3	FC2, F2	8	FEF	right	58.07
6, 5	FC1, FCz	6	SMA	left - medial	73.21
7, 5	FC2, FCz	6	SMA	right - medial	62.95
9, 7	Cz, C1	6	SMA	left - medial	56.45
9, 8	Cz, C2	6	SMA	right - medial	55.39
10, 6	C4, FC4	6	PMC	right - lateral	56.87
6, 7	FC1, C1	6	PMC	left - lateral	81.78
7, 8	FC2, C2	6	PMC	right - lateral	82.46
8, 4	C3, FC3	6	PMC	left - lateral	61.71
11, 9	CP1, CPz	1, 5, 7	SSC	left	86.14
11, 10	CP1, P1	5, 7	SSC	left	92.32
12, 9	CP2, CPz	1, 3, 5, 7	SSC	right	91.65
12, 11	CP2, P2	5, 7	SSC	right	92.64
13, 10	Pz, P1	7	SSC	left	91.59
13, 11	Pz, P2	7	SSC	right	93.86

Note. Optode locations were specified in the fNIRS Optodes' Location Decider (fOLD) toolbox, using the Brodmann atlas. Minimum specificity level for channel inclusion was set at 50% in accordance with Pelicioni et al. (2022)[3]. PFC = prefrontal cortex; FEF = frontal eye fields; SMA = supplementary motor cortex; PMC = premotor cortex; SSC = somatosensory cortex.

3. Pelicioni PHS, Lord SR, Okubo Y, Menant JC (2022) Cortical activation during gait adaptability in people with Parkinson's disease. Gait Posture 91:247–253. https://doi.org/10.1016/j.gaitpost.2021.10.038

Table S2 Test-retest reliability based on average ICCs for relative HbO_2 levels during the postural task for total, left and right ROIs

		Mean ± SD (μmol/L)			ICC (95% CI)		SEM (μmol/L)	
		Test 1	Test 2	Test 3	Test 1-2	Test 1-3	Test 1-2	Test 1-3
PFC	total	1.78 ± 8.28	1.53 ± 6.04	2.92 ± 6.02	0.93 (0.83, 0.97)**	0.67 (0.15, 0.87)*	1.89	4.19
	left	2.39 ± 10.47	1.12 ± 7.61	1.94 ± 6.58	0.87 (0.67, 0.95)**	0.80 (0.48, 0.92)**	3.34	3.94
	right	1.17 ± 7.78	1.94 ± 6.48	3.90 ± 6.78	0.91 (0.78, 0.67)**	0.00 (0.00, 0.56)	2.14	7.30
FEF	total	1.15 ± 4.63	1.70 ± 3.05	1.58 ± 3.50	0.68 (0.18, 0.87)**	0.78 (0.44, 0.91)**	2.23	1.93
	left	1.40 ± 4.47	1.77 ± 4.16	0.72 ± 4.65	0.65 (0.11, 0.86)*	0.47 (0.00, 0.79)	2.54	3.33
	right	0.90 ± 5.98	1.63 ± 4.13	2.45 ± 4.02	0.74 (0.35, 0.90)**	0.65 (0.15, 0.86)*	2.61	3.00
SMA	total	2.15 ± 2.79	1.42 ± 5.57	3.20 ± 5.74	0.65 (0.10, 0.86)*	0.02 (0.00, 0.62)	3.37	5.71
	left	1.82 ± 6.55	1.73 ± 5.90	3.43 ± 5.67	0.66 (0.12, 0.87)*	0.36 (0.00, 0.75)	3.36	4.89
	right	2.48 ± 5.97	1.11 ± 6.72	2.98 ± 6.91	0.58 (0.00, 0.83)*	0.00 (0.00, 0.60)	4.14	6.45
PMC	total	4.08 ± 5.95	3.68 ± 4.77	5.05 ± 5.30	0.88 (0.69, 0.95)**	0.00 (0.00, 0.24)	1.87	5.63
	left	4.16 ± 7.29	3.62 ± 5.73	5.11 ± 6.35	0.85 (0.62, 0.94)**	0.00 (0.00, 0.17)	2.55	6.83
	right	4.00 ± 5.69	3.75 ± 5.29	5.00 ± 5.53	0.85 (0.63, 0.94)**	0.01 (0.00, 0.62)	2.11	5.59
SSC	total	3.54 ± 3.75	2.40 ± 4.26	3.20 ± 4.45	0.88 (0.67, 0.95)**	0.68 (0.17, 0.87)*	1.42	2.34
	left	2.98 ± 4.07	2.37 ± 3.85	3.29 ± 5.46	0.75 (0.37, 0.90)**	0.12 (0.00, 0.66)	1.98	4.52
	right	4.09 ± 5.41	2.43 ± 5.27	3.12 ± 5.34	0.85 (0.60, 0.94)**	0.79 (0.47, 0.92)**	2.10	2.47

Note. HbO_2 = oxygenated hemoglobin; ROI = region of interest; SD = standard deviation; ICC = intraclass correlation coefficient; SEM = standard error of measurement; PFC = prefrontal cortex; FEF = frontal eye fields; SMA = supplementary motor area; PMC = premotor cortex; SSC = somatosensory cortex. *Significant at $\alpha<0.05$. **Significant at $\alpha<0.01$.

Table S3. Test-retest reliability based on average ICCs for relative HHb levels during the postural task for total, left and right ROIs

		Mean ± SD (μmol/L)			ICC (95% CI)		SEM (μmol/L)	
		Test 1	Test 2	Test 3	Test 1-2	Test 1-3	Test 1-2	Test 1-3
PFC	total	0.97 ± 3.24	0.56 ± 2.98	0.88 ± 3.31	0.64 (0.07, 0.86)*	0.76 (0.38, 0.91)**	1.88	1.61
	left	0.64 ± 3.93	7.54 ± 4.36	1.05 ± 3.60	0.46 (0.00, 0.79)	0.54 (0.00, 0.82)	3.04	2.54
	right	1.31 ± 3.46	0.37 ± 2.24	0.72 ± 3.28	0.77 (0.44, 0.91)**	0.84 (0.60, 0.94)**	1.39	1.36
FEF	total	0.57 ± 3.21	0.03 ± 1.55	0.25 ± 1.74	0.54 (0.00, 0.82)	0.56 (0.00, 0.83)*	1.71	1.71
	left	-0.056 ± 1.48	0.22 ± 1.65	0.27 ± 2.19	0.45 (0.00, 0.79)	0.50 (0.00, 0.81)	1.16	1.32
	right	1.20 ± 5.64	-0.15 ± 2.14	0.24 ± 1.91	0.62 (0.07, 0.85)*	0.44 (0.00, 0.78)	2.65	3.15
SMA	total	-0.31 ± 3.78	-0.74 ± 2.52	-0.10 ± 2.28	0.44 (0.00, 0.78)	0.31 (0.00, 0.74)	2.41	2.57
	left	-0.53 ± 4.29	-0.62 ± 2.69	-0.12 ± 2.80	0.40 (0.00, 0.77)	0.40 (0.00, 0.77)	2.76	2.81
	right	-0.08 ± 3.65	-0.86 ± 2.59	-0.09 ± 2.43	0.47 (0.00, 0.79)	0.25 (0.00, 0.71)	2.31	2.69
PMC	total	0.26 ± 2.26	-0.44 ± 2.52	0.14 ± 2.28	0.91 (0.73, 0.97)**	0.28 (0.00, 0.72)	0.73	1.93
	left	0.14 ± 3.23	-0.26 ± 3.52	0.27 ± 2.63	0.93 (0.82, 0.97)**	0.00 (0.00, 0.62)	0.91	2.94
	right	0.37 ± 1.86	-0.61 ± 2.18	0.01 ± 2.60	0.82 (0.40, 0.94)**	0.58 (0.00, 0.84)*	0.87	1.46
SSC	total	-1.00 ± 1.22	-1.28 ± 1.43	-1.08 ± 1.14	0.83 (0.57, 0.93)**	0.63 (0.03, 0.85)*	0.55	0.72
	left	-0.98 ± 1.66	-1.22 ± 1.66	-1.13 ± 1.56	0.89 (0.73, 0.96)**	0.58 (0.00, 0.83)*	0.55	1.05
	right	-1.01 ± 1.03	-1.34 ± 1.53	-1.03 ± 1.18	0.70 (0.29, 0.88)**	0.54 (0.00, 0.82)	0.70	0.75

Note. HHb = deoxygenated hemoglobin; ROI = region of interest; ICC = intraclass correlation coefficient; SEM = standard error of measurement; PFC = prefrontal cortex; FEF = frontal eye fields; SMA = supplementary motor area; PMC = premotor cortex; SSC = somatosensory cortex. *Significant at $\alpha<0.05$. **Significant at $\alpha<0.01$.

Table S4. Test-retest reliability based on average ICCs for relative HbO_2 and HHb levels during the finger tapping task

	Mean ± SD (µmol/L)			ICC (95% CI)		SEM (µmol/L)	
	Test 1	Test 2	Test 3	Test 1-2	Test 1-3	Test 1-2	Test 1-3
HbO	5.40 ± 3.38	4.97 ± 3.46	6.60 ± 4.04	0.79 (0.48-0.92)**	0.55 (0.00-0.82)*	1.56	2.49
HHb	-1.73 ± 1.46	-1.58 ± 1.28	-2.11 ± 2.05	0.89 (0.72-0.96)**	0.79 (0.48-0.92)**	7.23	8.68

Note. Values are determined for the task-specific hand motor channel C3-C1. HbO_2 = oxygenated hemoglobin; HHb = deoxygenated hemoglobin; SD = standard deviation; ICC = intraclass correlation coefficient; SEM = standard error of measurement. *Significant at $\alpha<0.05$. **Significant at $\alpha<0.01$.
